# Validation of a Questionnaire on Sexual and Reproductive Health Among Immigrant Vocational Education Students in Portugal from São Tomé and Príncipe

**DOI:** 10.1007/s10900-023-01230-8

**Published:** 2023-05-09

**Authors:** Cátia Brazete, Diogo Caveiro, Marisa Lopes Neto, João Pedro Dinis, Luís Castro Rocha, Lígia Sá, Ricardo Carvalhido

**Affiliations:** 1 Unidade Local de Saúde do Alto Minho, Unidade de Saúde Pública, Viana do Castelo, Portugal; 2 Universidade do Porto, Instituto de Saúde Pública, Porto, Portugal; 3 SWITCH ON – Academia de Formação Profissional, Viana do Castelo, Portugal; 4 Escola Superior de Educação, Instituto Politécnico de Viana do Castelo, Viana do Castelo, Portugal

**Keywords:** Sexual health, Contraception, School Health, Minority Health

## Abstract

Sexual and reproductive health interventions’ effectiveness should be evaluated using a valid instrument. This study aimed to validate a questionnaire on sexual and reproductive health in adolescents and young adults from São Tomé and Príncipe who voluntarily enrolled in a vocational school in Portugal to complete their 12th school year and obtain a professional card.

A questionnaire consisting of perception and knowledge sections was adapted from previous questionnaires. We followed the steps of psychometric analysis. Firstly, we assessed face validity and content validity. We used factorial analysis to validate the perceptions section (which included Likert-type questions). For the knowledge section (which consisted of multiple-choice questions), we used the key check, discrimination index, and difficulty index. We evaluated internal consistency through Cronbach’s alpha for the perceptions section and the Kuder-Richardson score for the knowledge section.

Out of 105 invited students, a total of ninety students were enrolled in this study, of whom 88 completed most of the questions. The exploratory analysis showed that most students agreed with the right to refuse intercourse. However, while approximately 23% disagreed that consensual sex among adult women or men is always wrong, around the same proportion agreed. A considerable number of students recognized condoms and pills as effective methods of contraception. However, most students were unfamiliar with other methods. Most knowledge questions showed acceptable difficulty levels, and the discrimination index varied among questions. The knowledge questions demonstrated good consistency levels. This study enabled us to develop an appropriate instrument for evaluating the effectiveness of public health interventions and identifying specific knowledge gaps in migrant populations from low-income countries. This will help prioritise topics to be addressed in sexual health education sessions.

**Supplementary Information:**

The online version contains supplementary material available at 10.1007/s10900-023-01230-8.

## Introduction

It is well-known that school-based sex education can prevent unwanted pregnancies, and promote positive sexual health at the individual, family, community, or health system level [1–3]. Most adolescents do not feel comfortable discussing sexuality issues with their parents [4]. Moreover, they spend most of their time at school, which is why schools play a vital role in conveying information and demystifying false beliefs related to sexual and reproductive health.

A substantial part of the world’s youth lives in developing countries. However, little is known about their knowledge and understanding of sexual health [5–7]. Adolescent pregnancy is a significant public health problem in São Tomé and Príncipe [8]. It has one of the highest adolescent pregnancy rates in Sub-Saharan Africa, reaching around 27% [9]. Teenage pregnancy can have adverse consequences for the mother, such as obstetric complications, early school dropout, low levels of education, less skilled jobs, and a higher probability of depending on social allowances, exacerbating the low socioeconomic status of people from developing countries [10].

The Sustainable Development Goals established by the United Nations emphasize the significance of advancing the eradication and treatment of sexually transmitted infections (STIs). STIs are among the most prevalent diseases globally, with Sub-Saharan Africa having the highest prevalence [11]. Adolescents have some of the highest incidence rates of STIs [12]. Studies have shown the effectiveness of school-based interventions in preventing STIs among young people [13, 14].

A pilot study is a type of feasibility study performed in preparation for a major study [15]. Conducting a pilot study provides information on whether the survey instrument is appropriate or too complex for the participants. A well-conducted pilot study can benefit other projects that use similar methods and tools. Well-designed and well-performed pilot studies can provide information about the research process and, in some cases, insights into the expected results [7]. A few studies report on the results of questionnaire validation in sexual and reproductive health among migrants from low-income countries.

SWITCH ON is a vocational academy for students who have completed their 9th year. One of its schools is in Viana do Castelo, a city in northern Portugal, where there are a total of 270 students, most of them from São Tomé and Príncipe, a Portuguese-speaking African country. These students are fundamental in the current scenario of the country’s systemic shortage of workforce in the construction and energy sector, which has a marked tendency to intensify over the next decade, forcing the country to resort to migrants.

This study aims to validate an adapted questionnaire about sexual and reproductive health for a population of adolescents and young adults from São Tomé and Príncipe. The secondary objectives were to analyse factors associated with adolescents’ perceptions and knowledge related to sexual and reproductive health and to identify gaps to prioritize topics to be addressed in a public health intervention.

## Methods

This study was designed as part of a field study. We selected five courses, comprising a total of 105 students, who were having classes in the school between December 2022 and February 2023, the period of the pre-intervention test, health education sessions, and post-intervention test. We performed a cluster-randomized sampling, randomly allocating each course to one of three groups: (A) health education sessions (two courses), (B) written information delivery (two courses), and (C) control group without intervention (one course).

The inclusion criteria were being a student between the ages of 15 and 26, coming from a Portuguese-speaking country, residing in Viana do Castelo, and undergoing training at the SWITCH ON Vocational Training Academy. Students who did not provide informed consent or who, for some reason, were not present in the classroom when completing the questionnaires were excluded.

A pre-intervention pilot questionnaire on perceptions and knowledge related to sexual and reproductive health was administered to all participants. It consisted of questions from three previous questionnaires: (1) sociodemographic questions and questions from the perceptions section of an internationally validated standard questionnaire [24], (2) questions about knowledge of sexual and reproductive health from previous studies conducted in Portugal [16, 17] and validated for the Portuguese population [17]. Our questionnaire mainly comprises closed-ended questions.

The questionnaire is divided into four sections: (1) presentation of the study, and consent to complete and process data from the questionnaire, (2) sociodemographic questions, (3) perceptions about sexuality (Likert-style questions), and (4) knowledge of sexual and reproductive health (multiple-choice questions (MCQ), with one or more correct answers). Sociodemographic variables were selected because they are potential determinants of students’ knowledge of sexual and reproductive health and could be analysed as potential confounders. The questionnaire was administered by the main investigator and self-completed by students in the classroom using Microsoft Forms®.

To assess face validity, the method of spoken reflection was used. We first delivered the questionnaire to six randomly selected adolescents, who were representative of the desired sample. Then, we conducted face-to-face interviews to assess the items’ difficulty, relevance, and ambiguity. Based on their feedback, we made the necessary modifications [16].

The content validity was assessed qualitatively by three experts who ascertained whether the questions were relevant, appropriate, and representative of the construct being examined [18]. The items were subjected to exploratory analysis, and the respective results are summarized in the Results section. Knowledge questions that were not answered were considered incorrect.

Construct validity was evaluated separately for the perceptions and knowledge sections using different methodologies. We used exploratory factor analysis (principal component analysis with varimax rotation) for perception questions. The Kaiser-Meyer-Olkin (KMO) measure of sampling adequacy and Bartlett’s Test of Sphericity were used to assess the suitability of the sample for factor analysis [19]. The knowledge section includes dichotomous items that were not subjected to factor analysis. Instead, we performed a key check, item discrimination analysis, and item difficulty analysis, following the methodology proposed by Considine et al. [20]. The key check determines whether the correct answer to the MCQ is correct and verifies that there is only one answer that can be considered valid [20]. The questionnaire was filled out by three medical doctors, and questions whose answers raised doubts were confirmed by a senior doctor who is an expert in sexual and reproductive health [18]. Item discrimination analysis examines how each MCQ is related to the overall test score and is computed using a biserial point correlation [20, 21].

The most common formula used to measure internal consistency is ‘coefficient alpha’ [20] or ‘Cronbach’s alpha score’. This was used to analyse the internal consistency of the perceptions section. Kuder-Richardson (KR-20) is a special form of the coefficient alpha formula, used for dichotomous data, such as answers that are scored as correct or incorrect in the context of MCQs [22]. It is interpreted like the Cronbach alpha scores: the questionnaire is considered consistent if the score is higher than 0.7. We used the KR-20 score to analyse the internal consistency of the knowledge section. Those students with knowledge scores above the mean were categorized as having good knowledge, while those with knowledge scores below the mean were categorized as having poor knowledge. We searched for associations between the sociodemographic variables, and the knowledge test score (as a continuous and categorical variable).

The data were analysed using R software, version 4.1.3 for RStudio Build 461, Vienna, Austria, and IBM SPSS Statistics for Windows, version 27.0, Armonk, USA.

The study protocol was approved by the Ethics Committee for Health of the Local Health Unit of Alto Minho, with reference number 78/2022.

Out of 105 invited students, a total of 90 students were enrolled in this study, of whom 88 completed most of the questions. Their characteristics can be found in Table 1. The median age was 21 (SEM: 0.303), 70% were female, and 38% had received education on sexual and reproductive health in the last two years.

## Results

### Sociodemographic Variables

Among the female students, 12 (20%) reported at least one previous pregnancy occurring between 16 and 21 years of age. When asked if they were willing to have a baby when they got pregnant, five (42%) answered ‘I did not want to get pregnant at that point’, and only one (8%) answered ‘I really to get pregnant at that time’.

**Table 1:** Sociodemographic variables descriptive analysis among vocational education students

Characteristics	N (%)	Median ± SEM
Age	79	21.0 ± 0.3
Sex at birth	87	
Male	26 (29.5)	
Female	61 (70.1)	
Intersex, indeterminate, or other sex	0 (0.0)	
I prefer not to answer	0 (0.0)	
Gender	88	
Boy/man	26 (29.5)	
Girl/woman	62 (70.5)	
Another way (please specify)	0 (0.0)	
Other	0 (0.0)	
Education level of legal guardians	88	
Primary school (4th grade)	4 (4.8)	
Basic secondary school (9th grade)	21 (25.0)	
Pre-university school (12th grade)	45 (53.6)	
Higher education (bachelor, master, doctor)	14 (16.7)	
Never attended school	0 (0.0)	
Sex education at school in the previous 2 years	87	
Yes	33 (37.9)	
No	38 (43.7)	
I do not remember	14 (16.1)	
Not applicable (I was not at school in the last 2 years)	2 (2.3)	

## Perceptions

### Exploratory Analysis

Answers to questions on perceptions regarding sexual and reproductive health can be found in Table 2. Most of the students agreed or strongly agreed that women (85%), and men (79%) have the right to refuse sexual intercourse if they do not want to engage in it. However, while 23% disagreed that consensual sex among adult women or men is always wrong, around the same proportion either agreed or had a neutral opinion (36–40%). Thirty-six per cent agreed with the right to abortion, and 64% believed that the couple should make this decision together.

**Table 2:** Perceptions on sexual and reproductive health among vocational education students (N = 88)

Item	Strongly disagree N (%)	Disagree N (%)	Do not agree nor disagree N (%)	Agree N (%)	Strongly agree N (%)	Missing N (%)
Sex education promotes sexual activity among adolescents and young people.	2 (2.3)	12 (13.6)	27 (30.7)	39 (44.3)	3 (3.4)	5 (5.7)
A woman has the right to say ‘no’ to sexual intercourse if she does not want to do it.	2 (2.3)	0 (0.0)	1 (1.1)	39 (44.3)	40 (45.5)	6 (6.8)
A man has the right to say ‘no’ to sexual intercourse if he does not want to do it.	0 (0.0)	2 (2.3)	1 (1.1)	44 (50.0)	29 (33.0)	12 (13.6)
It is acceptable for a woman to have sex before marriage.	3 (3.4)	15 (17.0)	28 (31.8)	30 (34.1)	4 (4.5)	8 (9.1)
It is acceptable for a man to have sex before marriage.	2 (2.3)	11 (12.5)	28 (31.8)	26 (29.5)	4 (4.5)	17 (19.3)
Having pleasurable sex is important to a woman’s sex life and overall well-being.	1 (1.1)	2 (2.3)	10 (11.4)	45 (51.1)	20 (22.7)	10 (11.4)
Having pleasurable sex is important to a man’s sex life and overall well-being.	0 (0.0)	1 (1.1)	13 (14.8)	43 (48.9)	20 (22.7)	11 (12.5)
Consensual sex between two adult women is always wrong.	18 (20.5)	12 (13.6)	28 (31.8)	16 (18.2)	3 (3.4)	11 (12.5)
Consensual sex between two adult men is always wrong.	17 (19.3)	9 (10.2)	29 (33.0)	17 (19.3)	1 (1.1)	15 (17.0)
Men naturally have greater sexual needs than women.	1 (1.1)	15 (17.0)	19 (21.6)	35 (39.8)	9 (10.2)	9 (10.2)
It is okay for a woman to use a modern contraception method to avoid or delay pregnancy if she wants to.	1 (1.1)	7 (8.0)	7 (8.0)	43 (48.9)	21 (23.9)	9 (10.2)
It is okay for a woman to have an abortion/termination if she does not want to have a child.	9 (10.2)	15 (17.0)	21 (23.9)	33 (37.5)	3 (3.4)	7 (8.0)

### Construct Validity and Internal Consistency Analysis

**Fig. 1:**
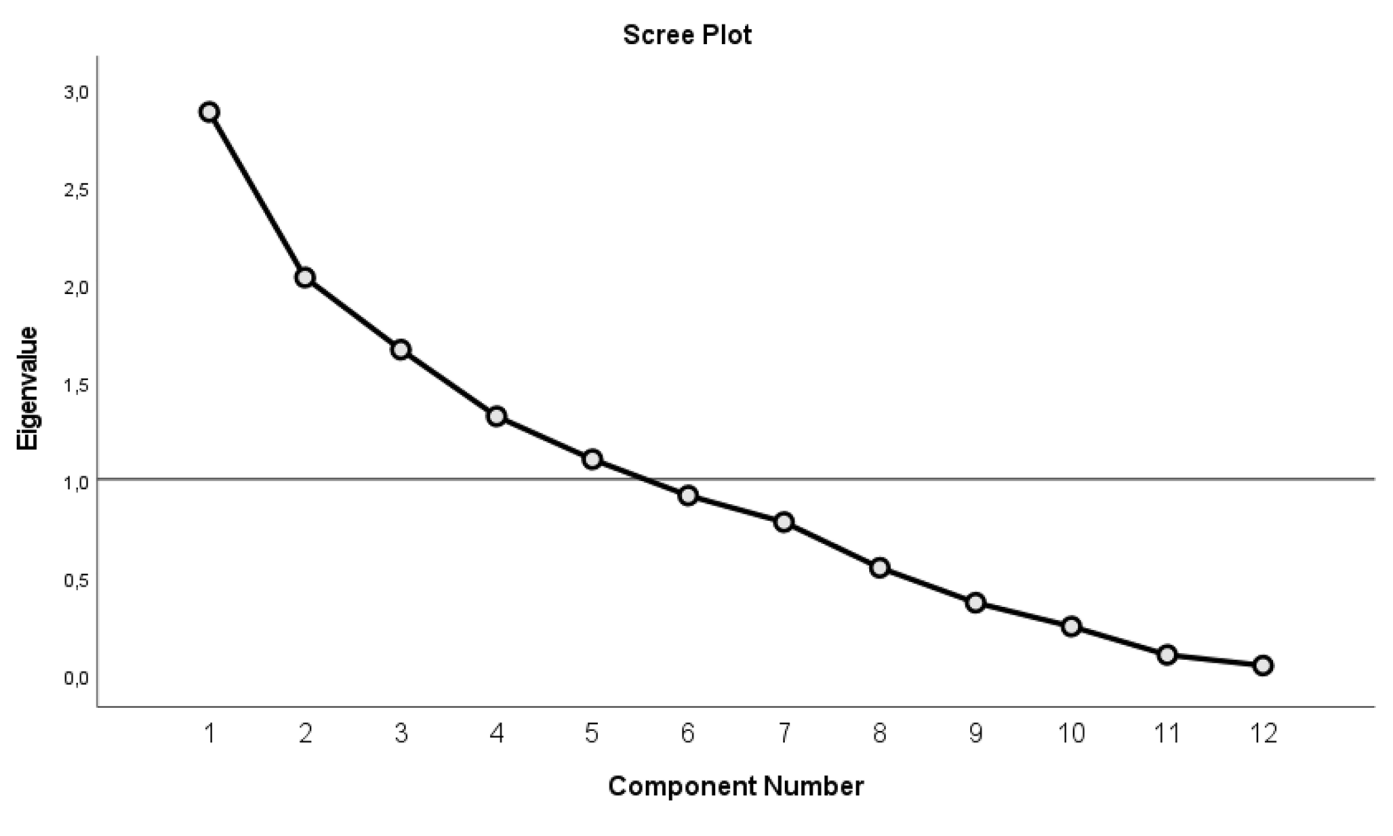
Scree plot of exploratory factorial analysis of perception questions

Based on Bartlett’s test, the correlation matrix significantly differed from the identity matrix, and the Kaiser-Meyer-Olkin measure confirmed its adequacy for principal component analysis. We performed a principal component analysis with varimax rotation, which revealed that the first principal component explained 24% of the variance and the second component accounted for 17% of the initial variance. According to the criterion of eigenvalue greater than 1, we retained five principal components, as shown in Fig. 1, which explained 75% of the total variance.

The questions ‘A woman has the right to say ‘no’ to sexual intercourse if she does not want to do it’, ‘It is acceptable for a woman to have sex before marriage’, ‘Having pleasurable sex is important to a woman’s sex life and overall well-being’, and ’Men naturally have greater sexual needs than women’ were considered redundant and removed. The calculated coefficient alpha was 0.34. It was not possible to conduct a confirmatory factor analysis using the usual method, due to our small sample size.

## Knowledge

### Exploratory Analysis

The questions on knowledge and their corresponding answers can be found in Table 3. The mean score for knowledge was 10.93 (± 2.47) out of 20 points (54%). Most students (89%) accurately defined basic reproductive concepts, such as puberty, and recognised condoms as a contraceptive agent (91%). However, 88% of the students were unfamiliar with the vaginal ring, and 79% were not familiar with the intrauterine device. Moreover, some misconceptions were observed regarding condom use, as 88% believed that condoms should be put on only at the time of ejaculation. Knowledge gaps were also identified regarding sexually transmitted infections (STIs), as 88% and 83% of participants did not recognise gonorrhoea and hepatitis B as STIs respectively. Additionally, the students lacked insight into what to do in case a condom breaks: 91% of participants did not know that emergency contraception can be used up to five days after unprotected intercourse, 84% did not know that emergency contraception is to be used on occasion, and 71% did not know that, in case of a broken condom, the woman should not douche but instead wash and urinate as soon as possible.

**Table 3:** Knowledge of sexual and reproductive health among vocational education students

Question	Correct N (%)	Incorrect N (%)
**The set of changes that occur in adolescence is called:**	80 (89%)	10 (11%)
**What causes physical and psychological changes in adolescence?**	50 (56%)	40 (44%)
**Age at which puberty begins in women and men**	69 (77%)	21 (23%)
**Ovulation is:**	38 (42%)	52 (58%)
**The fertile period in women**	26 (29%)	64 (71%)
**The fertile period in men**	33 (37%)	57 (63%)
**Of the following methods of contraception, choose the ones you know:**		
Contraceptive ring	11 (13%)	77 (87%)
Condom	80 (91%)	8 (9%)
Pill	62 (70%)	26 (30%)
Intrauterine device/Intrauterine system	18 (20%)	70 (80%)
Subdermal implant	23 (26%)	65 (74%)
**Which of the following statements do you consider to be correct:**		
A girl can get pregnant the first time she has sex.	76 (86%)	12 (14%)
If the girl does not enjoy sexual intercourse, she does not get pregnant.	85 (97%)	3 (3%)
Orgasm happens to both men and women.	34 (39%)	54 (61%)
A girl is at risk of becoming pregnant without full insertion of the penis.	41 (47%)	47 (53%)
‘Pulling out’ is an effective method of contraception.	88 (100%)	0 (0%)
The condom is an effective method of contraception when used correctly and is the only one that protects against sexually transmitted infections.	53 (60%)	35 (40%)
Sperm cells can survive inside the vagina for about 72 h.	46 (52%)	42 (48%)
**Which of the following statements do you consider to be correct? You can choose more than one answer.**		
The condom can be put on at the moment of ejaculation.	83 (94%)	5 (6%)
If you have had sex without a condom just once, pregnancy is not possible.	77 (88%)	11 (13%)
Condoms correctly used during sexual intercourse prevent pregnancy and protect against sexually transmitted infections.	75 (85%)	13 (15%)
A condom can be safely reused.	68 (77%)	20 (23%)
Condoms decrease the pleasure of sexual intercourse.	56 (64%)	32 (36%)
**Which of the following statements do you consider to be correct? You can choose more than one answer.**		
**If the condom breaks, you must:**		
Take the penis out of the vagina immediately.	68 (77%)	20 (23%)
The woman should not douche but instead, wash and urinate as quickly as possible.	26 (30%)	62 (70%)
Apply a spermicide if possible.	85 (97%)	3 (3%)
Go to the primary care unit immediately or within 24 h after sexual intercourse.	53 (60%)	35 (40%)
**Which of the following infections do you consider to be sexually transmitted?**		
Gonorrhea	11 (13%)	77 (88%)
Syphilis	18 (20%)	70 (80%)
HIV/AIDS	84 (95%)	4 (5%)
Hepatitis B	14 (16%)	74 (84%)
Human Papilloma Virus (HPV)	31 (35%)	57 (65%)
Candidiasis	79 (90%)	9 (10%)
**Mark the statements you believe to be true:**		
A person can become infected with HIV/AIDS if they use a needle/syringe already used by someone else.	75 (85%)	13 (15%)
If someone infected with HIV/AIDS coughs or sneezes near other people, they could also become infected.	82 (93%)	6 (7%)
A man can become infected with HIV/AIDS if he has unprotected sex with another man.	32 (36%)	56 (64%)
If a woman infected with HIV/AIDS is pregnant, her baby can become infected.	27 (31%)	61 (69%)
A person can become infected with HIV/AIDS if he hugs someone infected.	86 (98%)	2 (2%)
The pill can protect a woman from contracting HIV/AIDS.	86 (98%)	2 (2%)
A person can become infected with HIV/AIDS if they have sex without using a condom, even just once.	62 (70%)	26 (30%)
A person can look very healthy and be infected with HIV.	53 (60%)	35 (40%)
A person can become infected with HIV/AIDS if they are bitten by an insect.	78 (89%)	10 (11%)
A person can become infected with HIV/AIDS by using eating or drinking utensils (plates, silverware, cups) already used by someone else.	79 (90%)	9 (10%)
**Regarding the use of the pill as a method of contraception, mark the statements that you consider to be true. You can choose more than one option.**		
It is a drug with a daily dose of 1 pill, around the same time of the day.	33 (38%)	55 (63%)
The pill acts on the menstrual cycle and acts on inhibiting ovulation.	23 (26%)	65 (74%)
It prevents the woman from becoming pregnant.	66 (75%)	22 (25%)
It maintains regular cycles and decreases menstrual pain.	14 (16%)	74 (84%)
The pill can protect a woman from sexually transmitted infections.	83 (94%)	5 (6%)
**Regarding the morning-after pill/emergency pill, do you consider that:**		
It can be taken up to 5 days after unprotected sexual intercourse.	8 (9%)	80 (91%)
The woman may feel nauseous or even vomit after taking the morning after/emergency pill.	30 (34%)	58 (66%)
The morning-after/emergency pill is a method of contraception.	49 (56%)	39 (44%)
The morning-after pill is to be used on occasion.	14 (16%)	74 (84%)
The morning-after pill can and should be used regularly.	70 (80%)	18 (20%)

### Construct Validity and Internal Consistency Analysis

As observed in Table 4, the item discrimination indices ranged between − 0.14 and 0.46, with an average of 0.19. The difficulty of the knowledge items varied from 0.11 to 0.94, with an average of 0.55. The KR20 score for the knowledge section was 0.73.

**Table 4:** Construct validation indexes and internal consistency scores for knowledge questions

Question	Mean	SD	Skew	Item difficulty index	Item discrimination index	KR- 20 score
16	0.89	0.32	-2.52	0.89	0.38	0.723
17	0.57	0.5	-0.27	0.57	0.20	0.728
18	0.76	0.43	-1.21	0.76	0.33	0.722
19	0.41	0.49	0.37	0.41	0.25	0.725
20	0.29	0.46	0.95	0.29	0.16	0.728
21	0.37	0.48	0.56	0.37	0.43	0.715
22. A	0.12	0.33	2.35	0.12	0.39	0.721
22. B	0.91	0.29	-2.94	0.91	0.34	0.724
22. C	0.69	0.47	-0.83	0.69	0.46	0.715
22. D	0.2	0.4	1.53	0.20	0.39	0.719
22. E	0.27	0.44	1.07	0.27	0.28	0.724
23. A	0.84	0.36	-1.93	0.84	0.24	0.727
23. C	0.4	0.49	0.42	0.40	0.32	0.732
23. D	0.47	0.5	0.14	0.47	0.41	0.720
23. E	0.87	0.34	-2.19	0.87	-0.01	0.715
23. F	0.61	0.49	-0.46	0.61	0.29	0.735
23. G	0.52	0.5	-0.09	0.52	0.21	0.724
24. A	0.93	0.25	-3.53	0.93	0.07	0.727
24. B	0.88	0.33	-2.35	0.88	-0.04	0.732
24. C	0.86	0.35	-2.06	0.86	0.41	0.736
24.D	0.78	0.42	-1.36	0.78	-0.11	0.720
24. E	0.63	0.48	-0.56	0.63	-0.10	0.741
25. A	0.77	0.43	-1.28	0.77	0.29	0.742
25. B	0.3	0.46	0.89	0.30	0.15	0.722
25. D	0.61	0.49	-0.46	0.61	0.24	0.731
26. A	0.14	0.35	2.06	0.14	0.06	0.733
26. B	0.2	0.4	1.53	0.20	0.28	0.725
26. D	0.16	0.36	1.93	0.16	0.22	0.732
26. E	0.36	0.48	0.61	0.36	0.11	0.724
26. F	0.88	0.33	-2.35	0.88	-0.12	0.727
27. A	0.86	0.35	-2.06	0.86	0.35	0.727
27. C	0.36	0.48	0.61	0.36	0.32	0.730
27.D	0.3	0.46	0.89	0.30	0.21	0.739
27. G	0.69	0.47	-0.83	0.69	0.04	0.722
27. H	0.59	0.49	-0.37	0.59	0.09	0.735
27. I	0.88	0.33	-2.35	0.88	-0.03	0.722
27. J	0.9	0.3	-2.71	0.90	-0.04	0.728
28. A	0.39	0.49	0.46	0.39	0.38	0.730
28. B	0.27	0.44	1.07	0.27	0.22	0.732
28. C	0.76	0.43	-1.21	0.76	0.40	0.736
28. D	0.16	0.36	1.93	0.16	0.10	0.734
28. E	0.94	0.23	-3.95	0.94	-0.08	0.736
29. A	0.11	0.32	2.52	0.11	0.03	0.736
29. B	0.33	0.47	0.72	0.33	0.24	0.717
29. C	0.56	0.5	-0.23	0.56	-0.14	0.727
29.D	0.16	0.36	1.93	0.16	0.34	0.717
29. E	0.8	0.4	-1.53	0.80	0.05	0.731
Mean				0.55	0.19	0.732

*SD – standard Deviation. Mean inter-item-correlation = 0.050 · Kuder-Richardson (KR20) = 0.73*

## Discussion

We observed that unwanted pregnancies, including adolescent pregnancy, were a concern in this student population. Additionally, most students believed that the abortion decision should be made by the couple, despite Portuguese legislation stating that the woman had the final say. A considerable proportion of students considered same-sex intercourse to always be wrong. It would have been important to collect information about the students’ religion, as it had been associated with homophobic attitudes and behaviours in previous studies [23].

A low overall knowledge score was found in this study, similar to other diagnostic tests administered to students [24]. This may be related to informal sources of information, such as their peers, who are often equally uninformed.

HIV/AIDS was widely known as an STI among students. However, most of the students did not know about some of the HIV transmission routes, such as men who have sex with men or vertical transmission. Additionally, most students did not recognise gonorrhoea, syphilis, HPV, and hepatitis B as STIs, which could lead them to not give importance to the symptoms or choose not to use condoms if they found that their partner was not HIV positive.

Regarding STI prevention knowledge, most students knew that condoms reduced the risk of contracting STIs, which was consistent with other studies [25–27]. However, we observed that many newer contraceptive methods, such as contraceptive rings, intrauterine devices/systems, and subdermal implants, which are available for free in the Portuguese NHS, were not known to these students.

Unlike other similar studies [25], the variables ‘birth sex’, ‘gender’, ‘age’, ‘age group’, ‘previous sex education (last two years)’ and ‘education of legal guardians’ were not predictors of the score on the knowledge test. This may be related to an insufficient sample size that did not allow us to demonstrate any of these associations.

The instrument consisted of four sections, of which two were thoroughly analysed in this study. The perceptions section showed low internal consistency. Low-reliability coefficients may indicate that the test is too short or that the multiple-choice questions have little in common. Therefore, they can be used as a set of questions but not as a scale for a singular construct. During the diagnostic test, some students reported that the questionnaire was too long, so we decided to remove this section. Nevertheless, since the main objective of our study was to assess the effectiveness of a public health intervention in increasing knowledge of sexual and reproductive health, this change could increase the acceptability of the questionnaire.

On the other hand, the knowledge section demonstrated acceptable internal consistency. Furthermore, most of the items showed adequate item difficulty. However, 27 items showed an unacceptable discrimination index (below 0.25). As a result, in the final questionnaire, we removed all items with discrimination indexes below 0.20 and between 0.20 and 0.25 with either too low (< 0.15) or too high (> 0.85) difficulty index.

Our study had some limitations, mainly due to its observational nature. The sample size was considered low, which might have reduced the statistical power and the generalizability of the results. A systematic review of articles available in Scopus recommended a sample size of 500–600 for questionnaire validation studies involving students [28]. Moreover, a proportion of students were not evaluated because they were out on internships. These students are mostly in their second year of the course, are probably older and may have different perceptions and/or knowledge regarding sexual and reproductive health. Furthermore, this study did not include vocational school dropouts who might have faced more challenges and risks related to sexual and reproductive health than the students who completed their education.

### Public Health Implications/Practice Implications

The poor knowledge scores on sexual and reproductive health shown in the questionnaire support the urgent need for health education interventions for these students. Additionally, it allowed the investigators to specifically identify the main knowledge gaps of the population in the study so that the topics to address in the intervention are prioritised. On the other hand, the validation of the pilot questionnaire contributed to improving its definitive version, with more valid and less ambiguous questions. In summary, the questionnaire can help educators, healthcare providers and policymakers understand the unique sexual and reproductive health needs of this population and develop targeted interventions to improve their overall health and well-being.

## Electronic Supplementary Material

Below is the link to the electronic supplementary material.

Supplementary Material 1
