# Supplementary material for: Validation of a Questionnaire on Sexual and Reproductive Health Among Immigrant Vocational Education Students in Portugal from São Tomé and Príncipe
Source: J Community Health. 2023 May 9;48(5):847–56. doi: 10.1007/s10900-023-01230-8 (PMC10469261; doi:10.1007/s10900-023-01230-8)
Supplement: Supplementary file 1 — Supplementary Material 1 [file 10900_2023_1230_MOESM1_ESM.docx]

Dear editor,

Dear reviewers,

Thank you for considering our article entitled *Validation of a questionnaire on Sexual and Reproductive Health among immigrant vocational education students in Portugal from São Tomé and Príncipe* to be possibly published in your journal. We appreciate the time and effort you used in reading and commenting on the article. Here are our responses, point by point, to reviewers’ comments. We are submitting two versions of the revised manuscript – one with track changes highlighted and the other clean.

Reviewer 1:

**This is an interesting study, but one that needs revision. The title needs to be revised to: Validation of a questionnaire on sexual and reproductive health among immigrant vocational education students in Portugal from Sao Tao and Principe.**

Thank you for your pertinent comment. The title was rewritten accordingly.

**The n of 105 needs to be stated in the Abstract and prominently in the Methods section.**

Thank you for your comment with which we fully agree. A sentence was added to the Abstract section – lines 43 and 44: “*Out of 105 invited students, a total of ninety students were enrolled in this study, of whom 88 completed most of the questions”*. Furthermore, that information was added to the beginning of the Methods section – lines 122 and 123 *- “We selected five courses, comprising a total of 105 students, that were having classes in school between December 2022 and February 2023 (…)”* and also to the end of the Methods section - lines 179 and 180 - “*Out of 105 invited students, a total of 90 students were enrolled in this study, of whom 88 completed most of the questions. (…)”.*

**English language and syntax need to be corrected throughout the manuscript.**

The English language was carefully revised and corrected throughout the manuscript, with final revision done by a professional English teacher and translator. We believe it is improved now and we are still available for further corrections if needed.

Reviewer 2:

**The columns in Table 3 are not aligned so that the numbers and percentages doo nor align with the statements. Need to correct. Give N in Table 2. The headings here need to be rearranged**.

Thank you for your comments. We improved and formatted the article, and the numbers and percentages are already aligned with the statements. Table 2 was updated including the N in the title. The headings were rearranged.

We hope that these alterations improved the article. We are available to proceed with further improvements if necessary.

We hope to hear from you soon,

Kind regards,

Cátia Brazete

(on behalf of all authors)
